# Human bite cases reported to health facilities in the Volta region of Ghana, 2019 to 2023

**DOI:** 10.3389/fpubh.2025.1575617

**Published:** 2025-11-04

**Authors:** Chrysantus Kubio, Maxwell Afetor, Samuel Adolf Bosoka, Williams Azumah Abanga, Victor Zeng, Christopher Sunkwa Tamal, Gyesi Razak lssahaku, Clement Tetteh Narh, Frank Baiden

**Affiliations:** ^1^Volta Regional Health Directorate, Ghana Health Service, Ho, Ghana; ^2^Department of Epidemiology and Biostatistics, Fred N. Binka School of Public Health, University of Health and Allied Sciences, Ho, Ghana; ^3^Health Information Unit, Volta Regional Health Directorate, Ghana Health Service, Ho, Ghana; ^4^Disease Surveillance Unit, Volta Regional Health Directorate, Ghana Health Service, Ho, Ghana; ^5^Saboba District Health Directorate, Ghana Health Service, Saboba, Ghana; ^6^WHO Country Office, Accra, Ghana; ^7^Laboratory Department, Tamale Teaching Hospital, Tamale, Ghana

**Keywords:** human bite, incidence, trend, Volta region, Ghana

## Abstract

**Introduction:**

Although human bite injuries occur less frequently than other types of injuries, they present notable public health challenges, yet they have received relatively little attention. This study examined the incidence, trend, and distribution of human bite injuries in the Volta Region of Ghana from 2019 to 2023.

**Method:**

A retrospective analysis of human bite injury data from the District Health Information Management System 2 (DHIMS-2) for the Volta Region was conducted for the period 2019 to 2023. Data on human bite injuries were obtained from the monthly outpatient (OPD) morbidity report form. Variables available and extracted were age, sex, district and year of report. The data was analyzed descriptively using Microsoft Excel and Quantum Geographic Information System (QGIS) and the findings presented in tables and graphs.

**Result:**

A total of 931 human bite injuries were reported to health facilities from 2019 to 2023, with the highest incidence of 12.0 per 100,000 recorded in 2021 and the lowest, 9.7 per 100,000 in 2019. Males and young adults between the ages of 18–34 accounted for 58.1 and 41.1%, respectively of all cases reported. Geographically, Ketu North recorded the highest number of cases, 184 (19.8%). There we no reported death due to human bite.

**Conclusion:**

Human bite injuries in the Volta Region have shown an upward trend over the years studied, with males and young adults aged 18 to 34 years affected in the majority of the reported cases. While the causes and infection outcomes were not determined, this study provides essential baseline data for further research into the causes and effects of these injuries.

## Background

Human bites are regarded as a significant public health issue around the globe. It is considered the third most common bite injury behind dog and cat bites (1). Human bites are often underestimated, but literature has it that they can lead to significant infections in the victims (2). Similar to monkey bites, human bites are generally more serious and more prone to infections and complications than those infected by other animals (3). Because there are more resident germs in the human mouth than in the mouths of dogs or cats, human bites usually infect the victim with more bacteria (4). Anaerobic bacteria often infect such wounds as a result of these bites (5). Findings from studies conducted in the United States have indicated that approximately 10% of human bites in infants would end in infection (6). With an increasing population, these bites can significantly increase with a pronounced rise in developing nations (7).

According to US estimates, one in two people will get bitten at some point in their lives, either by an animal or by a human (3). Human bites account for 3% of total bites seen at emergency departments (8). In some under-developed and developing countries in Africa, human bites are relatively common (9). However, studies conducted within these regions have primarily focused on human bites occurring at the oro-facial region of the human body (9–11). This inference could mean that most of these injuries occur in the head region. A study conducted across 12 African countries in 2020 on traumatic injuries revealed that out of a total of 200 cases seen, 51(25.5%) were as a result of human bites and these injuries occurred in the facial region (9). Most of these human bite injuries occurring in the orofacial region are a result of social conflicts (10). One of the two studies done on human bites in Ghana was in 2016 at the Komfo Anokye Teaching Hospital, which aimed to provide a general overview of orofacial human bites with a focus on etiology, presentations, anatomic locations, treatment, and outcome of treatment (11). Findings from the study showed that most of the offenders are known to the patients because they are rivals (11). Considering the fact that the incidence of human bite could increase due to potential rise in social conflicts, close contact activities and crowded living conditions (9), there is a need to review existing data to estimate the incidence, population at risk and identify patterns.

Human bites, although preventable, continue to represent a significant public health concern in both developing and developed regions. In Ghana, there has been insufficient research regarding the incidence, age distribution, and other demographic risk factors of human bite cases. Furthermore, there are currently no established national preventive or intervention strategies to address this issue. This situation underscores the urgent need for heightened public awareness and additional research. However, this will be based on baseline data. Therefore, this study aimed to examine the incidence, trends and geographic distribution of human bite cases reported in the Volta Region between 2019 and 2023.

## Methods

### Study design and setting

A retrospective analysis of aggregated secondary data was performed to describe human bite cases in the Volta Region of Ghana for the years 2019 to 2023. The Volta Region consist of 18 districts with an estimated population of 1.7 million people, with Ho as its capital city. The observed decrease in the Volta Region’s population between 2020 and 2021 is because the 2020 figure was a projection based on the 2010 census, while the 2021 figure comes from the actual 2021 census. The most populated district in the region is Ketu South Municipality, followed by Ho Municipality and Ketu North Municipality (Table 1). The predominant occupation in the region is farming, with most indigenes residing in rural communities. Fishing occurs along the coast and in communities along the Volta Lake (12). The Alavanyo-Nkonya conflict, which has been ongoing sporadically since 1923, is located in the Volta Region (13). The Volta Region currently has a total of 557 health facilities, with the majority of which are Community-based Health Planning Service (CHPS) compounds. The region has one Teaching Hospital, however, there are six districts without a primary hospital (14). The Volta Region was chosen for this review because there has not been any study on human bites in the region and ranks among the top 3 regions with the highest prevalence of domestic violence in Ghana (15). The region also experiences recurring community conflicts, such as the long-standing Alavanyo–Nkonya dispute, which reflects underlying social tension. Despite these conditions, there has been no prior study on human bite injuries in the region. Examining human bite patterns in this setting therefore provides important baseline information for understanding the burden and potential social drivers of such injuries in Ghana.

**Table 1:** Population by district/region, Volta Region, 2019 to 2023.

District/region	2019 annual population	2020 annual population	2021 annual population	2022 annual population	2023 annual population
Adaklu	44,307	45,325	38,649	39,461	40,289
Afadjato South	117,258	119,971	73,146	74,682	76,250
Agortime-Ziope	35,867	36,031	39,553	40,384	41,232
Akatsi North	41,691	42,655	32,541	33,224	33,922
Akatsi South	117,263	119,978	92,494	94,436	96,420
Anloga	97,713	99,966	94,895	96,888	98,922
Central Tongu	72,971	74,652	83,803	85,563	87,360
Ho	218,948	223,947	180,420	184,209	188,077
Ho West	117,268	119,963	82,886	84,627	86,404
Hohoe	133,021	136,090	92,211	93,760	95,729
Keta	84,696	86,649	78,862	80,518	82,209
Ketu North	122,546	125,366	114,846	117,258	119,720
Ketu South	198,051	202,614	253,122	258,438	263,865
Kpando	65,140	66,649	58,552	59,782	61,037
North Dayi	49,510	50,503	39,268	40,093	40,935
North Tongu	109,454	111,978	110,891	113,220	115,597
South Dayi	57,337	58,666	57,526	58,734	59,967
South Tongu	109,442	111,996	113,114	115,489	117,915
**Volta**	**1,792,483**	**1,832,999**	**1,636,779**	**1,670,764**	**1,705,849**

### Data collection and processing

All registered health facilities in the Volta Region have access to an electronic health record platform known as the District Health Information Management System-2 (DHIMS-2). This system facilitates the compilation of monthly data generated at each facility. When a client presents to a health facility with an injury, such as a human bite, demographic information, including age and sex, is recorded either electronically or in physical registers, along with the clinician’s diagnosis. At the end of each month, a summary of all new health conditions is prepared using the outpatient morbidity form, which is then entered into DHIMS-2 (13). For this study, the data visualization feature within DHIMS-2 was used to extract relevant information. As the data is reported by age and sex, the study was able to retrieve variables for age, sex, year, and the districts where human bite cases were documented and reported. To extract data on mortalities as a result of human bite, the Cause of Death (CoD) instance of the DHIMS was utilized. The event report page of the CoD instance was used to extract the number of deaths due to human bite.

### Data analysis

Data was analyzed descriptively based on person, place and time. The results were reported as frequencies and proportions. The QGIS software was used to geographically describe the distribution of the cases in the Volta Region. Incidence rate was calculated by dividing the number of cases reported by the total population within a specific year. Using estimates from the population and housing census, the age and sex specific populations were calculated. These estimates were used to compute the age and sex specific incidence (Table 2). District-specific incidence was also computed using the district-specific population by year (Table 1). The incidence was reported per 100,000 population with their respective 95% confidence intervals.

**Table 2:** Age and sex specific populations, Volta Region, 2019–2023.

Variables	Population				
	2019	2020	2021	2022	2023
Gender					
Male	855,014	874,340	780,743	796,955	813,690
Female	937,469	958,658	856,035	873,809	892,159
Age group					
0–4 yrs	198,966	203,463	181,682	185,455	189,349
5–9 yrs	213,305	218,127	194,777	198,821	202,996
10–14 yrs	200,758	205,296	183,319	187,126	191,055
15–17 yrs	127,266	130,143	116,211	118,624	121,115
18–19 yrs	77,077	78,819	70,381	71,843	73,352
20–34 yrs	408,686	417,924	373,185	380,934	388,934
35–49 yrs	277,835	284,115	253,701	258,968	264,407
50–59 yrs	121,889	124,644	111,301	113,612	115,998
60–69 yrs	87,832	89,817	80,202	81,867	83,587
70 + yrs	78,869	80,652	72,018	73,514	75,057

### Results

#### Characteristics of reported human bites

Over the 5 years, a total of 931 cases of human bites were recorded, of which 541 (58.1%) were males. There was no recorded mortality. In terms of age groupings, 321 (34.5%) of cases aged 20–34 years and 179 (19.2%) were between the ages of 35–49 years. Children less than 5 years old were 69 (7.4%). The majority of the cases were reported in 2023, 200 (21.5%). In terms of locations where these cases were reported, 184 (19.8%) human bites were recorded in Ketu North, 122 (13.1%) in Ho, and 85 (9.1%) in Akatsi North (Table 3).

**Table 3:** Characteristics of human bites reported to health facilities in the Volta Region, 2019–2023.

Variables	Year					Total = 931 N (%)
	2019 = 173 n (%)	2020 = 185 n (%)	2021 = 196 n (%)	2022 = 177 n (%)	2023 = 200 n (%)	
Gender						
Male	106 (61.3)	104 (56.2)	111 (56.6)	106 (59.9)	114 (57.0)	541 (58.1)
Female	67 (38.7)	81 (43.8)	85 (43.4)	71 (40.1)	86 (43.0)	390 (41.9)
Age group						
0–4	12 (6.9)	7 (3.8)	13 (6.6)	13 (7.3)	24 (12.0)	69 (7.4)
5–9	14 (8.1)	10 (5.4)	15 (7.7)	10 (5.6)	14 (7.0)	63 (6.8)
10–14	9 (5.2)	12 (6.5)	19 (9.7)	17 (9.6)	7 (3.5)	64 (6.9)
15–17	11 (6.4)	10 (5.4)	16 (8.2)	15 (8.5)	13 (6.5)	65 (7.0)
18–19	15 (8.7)	12 (6.5)	13 (6.6)	10 (5.6)	11 (5.5)	61 (6.6)
20–34	52 (30.1)	72 (38.9)	70 (35.7)	58 (32.8)	69 (34.5)	321 (34.5)
35–49	40 (23.1)	34 (18.4)	29 (14.8)	37 (20.9)	39 (19.5)	179 (19.2)
50–59	12 (6.9)	15 (8.1)	12 (6.1)	10 (5.6)	16 (8.0)	65 (7.0)
60–69	4 (2.3)	9 (4.9)	6 (3.1)	6 (3.4)	1 (0.5)	26 (2.8)
70+	4 (2.3)	4 (2.2)	3 (1.5)	1 (0.6)	6 (3.0)	18 (1.9)
District						
Adaklu	1 (0.6)	2 (1.1)	3 (1.5)	2 (1.1)	3 (1.5)	11 (1.2)
Afadjato South	9 (5.2)	5 (2.7)	7 (3.6)	13 (7.3)	15 (7.5)	49 (5.3)
Agortime-Ziope	1 (0.6)	1 (0.5)	2 (1.0)	4 (2.3)	3 (1.5)	11 (1.2)
Akatsi North	0 (0.0)	0 (0.0)	2 (1.0)	2 (1.1)	0 (0.0)	4 (0.4)
Akatsi South	14 (8.1)	10 (5.4)	29 (14.8)	14 (7.9)	18 (9.0)	85 (9.1)
Anloga	8 (4.6)	7 (3.8)	15 (7.7)	8 (4.5)	12 (6.0)	50 (5.4)
Central Tongu	2 (1.2)	6 (3.2)	4 (2.0)	5 (2.8)	9 (4.5)	26 (2.8)
Ho	43 (24.9)	21 (11.4)	22 (11.2)	16 (9.0)	20 (10.0)	122 (13.1)
Ho West	15 (8.7)	14 (7.6)	6 (3.1)	11 (6.2)	7 (3.5)	53 (5.7)
Hohoe	2 (1.2)	4 (2.2)	3 (1.5)	8 (4.5)	9 (4.5)	26 (2.8)
Keta	1 (0.6)	5 (2.7)	7 (3.6)	7 (4.0)	15 (7.5)	35 (3.8)
Ketu North	20 (11.6)	52 (28.1)	44 (22.4)	38 (21.5)	30 (15.0)	184 (19.8)
Ketu South	8 (4.6)	12 (6.5)	8 (4.1)	5 (2.8)	8 (4.0)	41 (4.4)
Kpando	5 (2.9)	8 (4.3)	3 (1.5)	3 (1.7)	2 (1.0)	21 (2.3)
North Dayi	1 (0.6)	2 (1.1)	5 (2.6)	3 (1.7)	8 (4.0)	19 (2.0)
North Tongu	13 (7.5)	18 (9.7)	12 (6.1)	11 (6.2)	7 (3.5)	61 (6.6)
South Dayi	13 (7.5)	11 (5.9)	7 (3.6)	11 (6.2)	16 (8.0)	58 (6.2)
South Tongu	17 (9.8)	7 (3.8)	17 (8.7)	16 (9.0)	18 (9.0)	75 (8.1)

#### Incidence of human bites in the Volta region, 2019–2023

**Figure 1:**
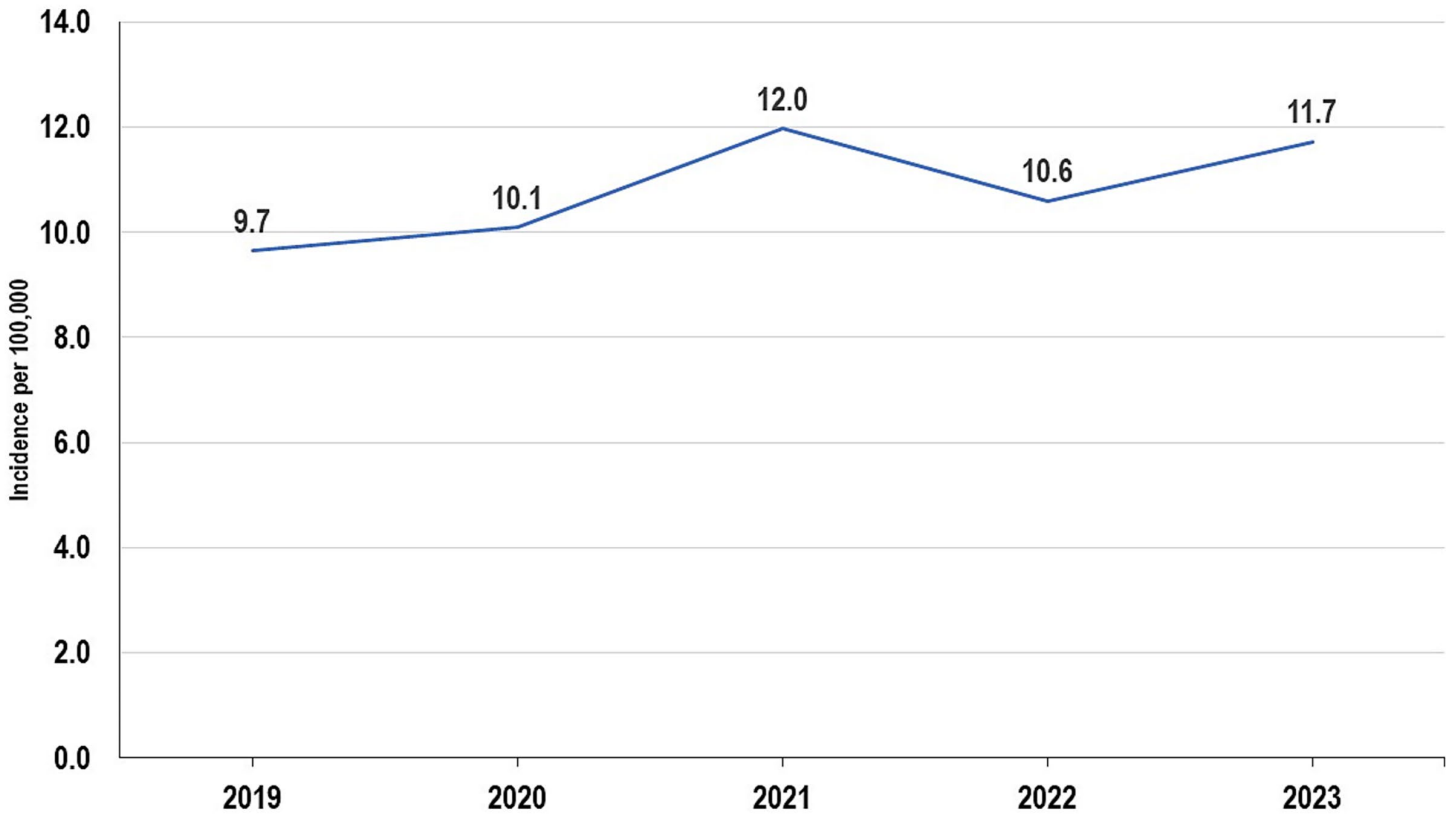
Trend of human bites in the Volta Region, 2019–2023.

**Figure 2:**
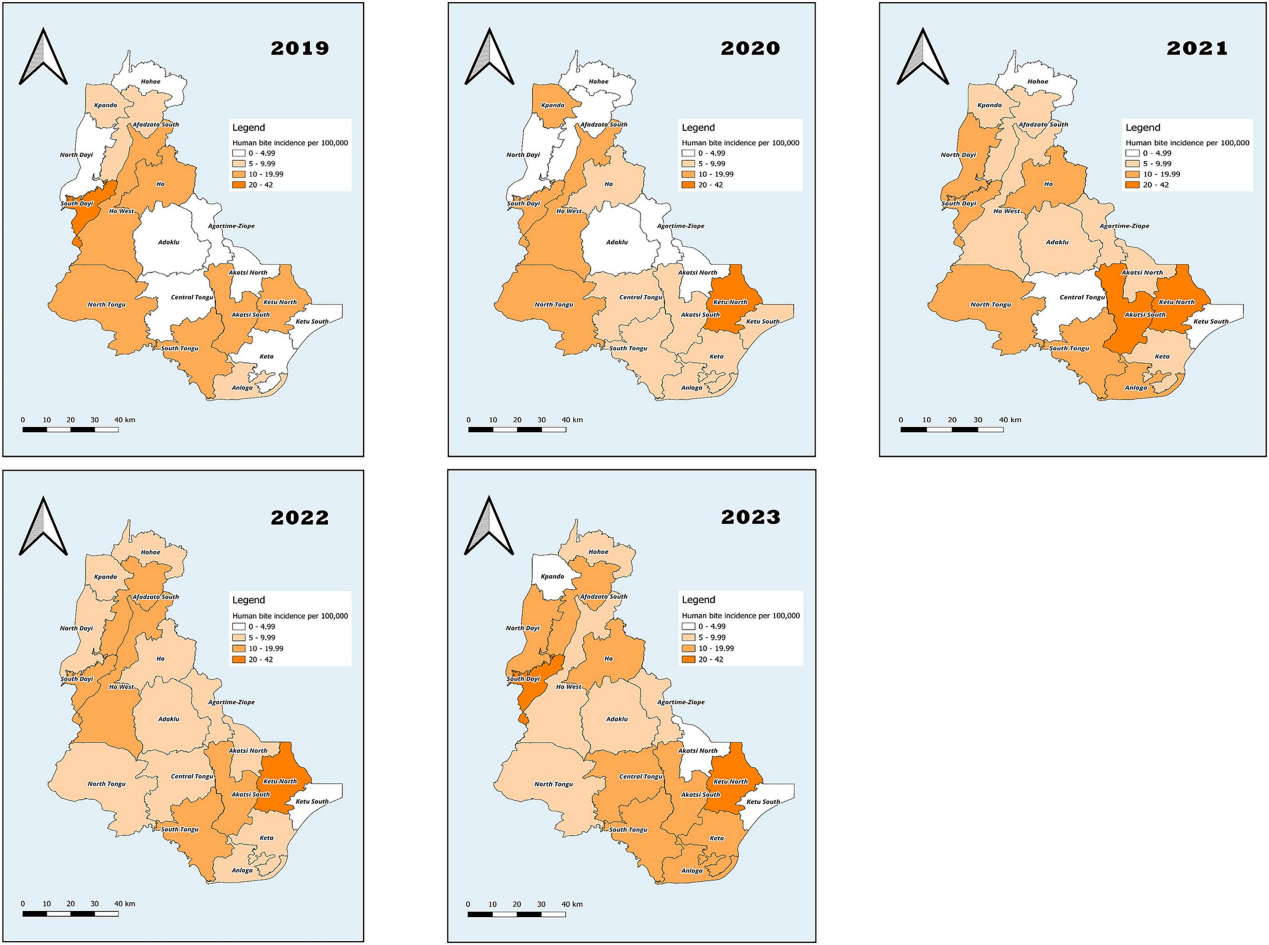
Human bites incidence by district in the Volta Region, 2019–2023.

In 2019, the incidence of human bites was 9.7 per 100,000 (Figure 1). The incidence was highest among males, 12.4 per 100,000 (95% CI: 10.0–14.8) and among persons aged 18–19 years, 19.5 per 100,000 (95% CI: 9.6–29.4; Table 4). Geographically, the incidence was highest in South Dayi, 22.7 per 100,000 (Figure 2). In 2020, the incidence of human bites increased to 10.1 per 100,000 (Figure 1). The incidence was lowest among females, 8.4 per 100,000 (95% CI: 6.6–10.2) but highest among persons aged 20–34 years, 17.2 per 100,000 (95% CI: 13.2–21.2; Table 4). Geographically, the incidence was highest in Ketu North, 41.5 per 100,000 (Figure 2). In 2021, the incidence further increased to 12.0 per 100,000 (Figure 1), resulting in a further increase in the incidence among persons aged 20–34 years, 18.8 (95% CI: 14.4–23.2; Table 4). There was, however, a decrease in incidence in Ketu North, 38.3 per 100,000, though it still remained the district with the highest incidence (Figure 2).

**Table 4:** Incidence of human bites by sex and age, in the Volta Region, 2019–2023.

Variable	2019	2020	2021	2022	2023
	Incidence per 100,000 (95% CI)	Incidence per 100,000 (95% CI)	Incidence per 100,000 (95% CI)	Incidence per 100,000 (95% CI)	Incidence per 100,000 (95% CI)
Gender					
Male	12.4 (10.0–14.8)	11.9 (9.6–14.2)	14.2 (11.6–16.8)	13.3 (10.8–15.8)	14.0 (11.4–16.6)
Female	7.1 (5.4–8.8)	8.4 (6.6–10.2)	9.9 (7.8–12.0)	8.1 (6.2–10.0)	9.6 (7.6–11.6)
Age Group					
0–4	6.0 (2.6–9.4)	3.4 (0.9–5.9)	7.2 (3.3–11.1)	7.0 (3.2–10.8)	12.7 (7.6–17.8)
5–9	6.6 (3.2–10.0)	4.6 (1.8–7.4)	7.7 (3.8–11.6)	5.0 (1.9–8.1)	6.9 (3.3–10.5)
10–14	4.5 (1.6–7.4)	5.8 (2.5–9.1)	10.4 (5.7–15.1)	9.1 (4.8–13.4)	3.7 (1.0–6.4)
15–17	8.6 (3.5–13.7)	7.7 (2.9–12.5)	13.8 (7.0–20.6)	12.6 (6.2–19.0)	10.7 (4.9–16.5)
18–19	19.5 (9.6–29.4)	15.2 (6.6–23.8)	18.5 (8.5–28.5)	13.9 (5.3–22.5)	15.0 (6.1–23.9)
20–34	12.7 (9.2–16.2)	17.2 (13.2–21.2)	18.8 (14.4–23.2)	15.2 (11.3–19.1)	17.7 (13.5–21.9)
35–49	14.4 (9.9–18.9)	12.0 (8.0–16.0)	11.4 (7.2–15.6)	14.3 (9.7–18.9)	14.8 (10.2–19.4)
50–59	9.8 (4.2–15.4)	12.0 (5.9–18.1)	10.8 (4.7–16.9)	8.8 (3.3–14.3)	13.8 (7.0–20.6)
60–69	4.6 (0.1–9.1)	10.0 (3.5–16.5)	7.5 (1.5–13.5)	7.3 (1.4–13.2)	1.2 (−1.1–3.5)
70+	5.1 (0.1–10.1)	5.0 (0.1–9.9)	4.2 (−0.5–8.9)	1.4 (−1.3–4.1)	8.0 (1.6–14.4)

In 2022, there was a decrease in the incidence of human bites, 10.6 per 100,000 (Figure 1). This affected the incidence among males, 13.3 per 100,00 (95% CI: 10.8–15.8) and also on persons aged 20–34 years, 15.2 (95% CI: 11.3–19.1; Table 4). In 2023, the incidence increased to 11.7 per 100,000 (Figure 1). As a result, there was an increase in incidence among males, 14.0 (95% CI: 11.4–16.6), which is the highest across the 5 years studied. The incidence among persons aged 20–34 years was 17.7 per 100,000 (13.5–21.9; Table 4). Geographically, the incidence was highest in South Dayi, 26.7 per 100,000 (Figure 2).

Compared to females, males had a significantly higher risk of human bite incidents (RR = 1.27, 95% CI: 1.11–1.44, *p* < 0.001). The risk of bites increased progressively with age, peaking among individuals aged 18–19 years (RR = 2.28, 95% CI: 1.59–3.27, *p* < 0.001) and 20–34 years (RR = 2.26, 95% CI: 1.74–2.98, *p* < 0.001), relative to children aged 0–4 years. Older adults (≥60 years) had a comparatively lower but non-significant risk. Overall, the results indicate that males and younger adults are at the greatest risk of human bite incidents (Table 5).

**Table 5:** Relative risk (RR) of human bites by sex and age.

Variables	RR (95% CI)	*p*-value
Sex		
Female	Ref	
Male	1.27 (1.11–1.44)	<0.001
Age Group		
0–4	Ref	
5–9	0.85 (0.59–1.22)	0.356
10–14	0.92 (0.64–1.31)	0.628
15–17	1.47 (1.03–2.10)	0.024
18–19	2.28 (1.59–3.27)	<0.001
20–34	2.26 (1.74–2.98)	<0.001
35–49	1.86 (1.40–2.49)	<0.001
50–59	1.54 (1.08–2.19)	0.012
60–69	0.85 (0.52–1.36)	0.491
70+	0.66 (0.37–1.12)	0.111

## Discussion

This study investigated the occurrence, trends, and patterns of human bites in the Volta Region of Ghana. A five-year analysis of human bites provided important insights into the incidence rates, demographic characteristics, and geographic disparities across the region. Findings from this study show that there seems to be an increase in the incidence of human bites over the years studied. Although the study did not establish the exact reason for the increase, surge in domestic violence in the region, may have contributed to this upward trend. The Ghana demographic and health survey indicated that the percentage of women aged 15–49 who have ever experienced domestic violence in the Volta region increased from 34.7% in 2008 to 40.3% in 2022. This places the Volta region in the top 3 regions with the highest prevalence (15).

This study offers important insights into the trends and demographic patterns of human bite cases in the Volta Region. However, the findings should be interpreted with caution because of the limitations of the DHIMS-2 data. Although DHIMS-2 captures routine health facility information, it lacks clinical and contextual details. It does not capture how the bite occurred, who the aggressor was, the body part affected, or the type and outcome of treatment. These gaps limit our ability to identify behavioral, social, and clinical factors that influence human bite incidents and their severity. Our study could only describe demographic and geographic patterns but could not assess causality or the risk of complications such as infections or hospital admissions. Despite these limitations, our study provides useful baseline data on the epidemiology of human bites in Ghana. The findings highlight the need to improve surveillance systems to include more detailed information. Enhancing DHIMS-2 to capture variables on injury mechanisms, treatment, and outcomes would support deeper analysis and better public health action. We recommend future studies should combine DHIMS-2 data with qualitative or hospital-based data to give a complete picture of the causes and effects of human bites in the region.

More males were affected compared to females. The district with the highest incidence is Ketu North and the lowest incidence was reported in Akatsi North. Although these estimates are lower than other diseases of public health concern within the region, there is a need to put in measures to curb the occurrence of these incidents, as human bites are generally more infectious than other animal bites (3). The plausible reasons for the differences in incidence across the districts could be related to population distribution. Districts such as Ketu North, which share borders with Togo, are more densely populated and experience higher cross-border trade and mobility. Increased population movement and social interaction in these settings may elevate the risk of interpersonal conflicts that lead to human bites. In contrast, smaller and more rural districts such as Akatsi North or Adaklu have lower population density and fewer social contact points, possibly explaining their lower incidence. In addition, variations in the strength of surveillance systems across districts may influence data accuracy, with some districts capturing and reporting cases more effectively than others. Although these explanations are speculative, they align with earlier reports linking human bites to domestic and social conflicts in African contexts (7, 10, 16). These findings further emphasize the need for further studies that explore the social and behavioral drivers of these injuries to design effective prevention strategies.

In our study, we reported a higher proportion of human bites among males compared to females. This is consistent with studies from Ghana and the US, which found that the majority of human bite victims are males (17–19). It is not surprising to see more males being victims of human bites because males are generally more aggressive and are involved in more risky behaviors such as fights, than females (20, 21). Also, in a physical altercation between a man and a woman, it is uncommon for the man to resort to biting. Typically, the woman is more likely to bite in self-defense. In contrast, studies conducted in several African countries, including Ghana, have found more females being victims of human bites (9–11, 22, 23). In the African settings where polygamy is practiced (24), co-wives often engage in physical altercations driven by jealousy, which can lead to one biting the other in an attempt to disfigure her rival (9). This may explain why studies have found a higher proportion of women being victims of human bites. The difference in estimates could be due to the fact that our study focused on all types of human bite injuries reported to the health facilities, whereas studies that reported higher proportions among females used data on persons who had orofacial human bite.

Our study found 34.5% of victims of human bites aged 20–34 years. In terms of incidence, the results show that persons aged 18–34 years are the most victims of human bites. This agrees with findings by Shubi et al., who found 45.5% of persons aged 20–29 years as victims of human bite (10). Also, Osaiyuwu & Osaguona found 38.5% of persons aged 28–37 years as victims of human bite (22). The high incidence among young adults correlates with the assertion that young adults are usually persons who are involved in conflicts, which results in physical altercation, because it is at this age that they begin intimate relationships (24). The relative risk analysis further supports these demographic trends. Males were about 27% more likely to experience human bite injuries than females, consistent with findings from previous studies in Ghana (11) and elsewhere (10) that associate aggressive behavior and interpersonal violence more commonly with men. Similarly, the risk peaked among young adults aged 18–34 years, the age group most likely to be involved in social or physical confrontations. These results suggest that targeted prevention and conflict resolution programs for young men could help reduce such incidents.

## Limitations and strengths

This study had important limitations. First, the DHIMS-2 data captured inadequate characteristics of human bite injuries. The cause and the affected areas were not routinely recorded. Furthermore, who did the biting, the type of treatment given and the outcome of the bite were not captured by the data source that was used for this analysis. The limited data variability restricted the level of analysis of the data for this study. Additionally, only human bite injuries reported at health facilities were included for this study, potentially underrepresenting the actual situation in the region. Despite these limitations, this study is the first to determine the rate, trend, and distribution of human bite injuries in the Volta Region of Ghana.

## Conclusion

The incidence of human bite injuries in the Volta Region appears to have risen over the years under study. While the incidence remains lower compared to other public health-related injuries in the region, it is essential to address this upward trend. Although our findings did not identify the specific causes of these injuries or their outcomes, we believe this study offers valuable baseline data for further research into the potential causes and outcomes of such injuries.

## Data Availability

The original contributions presented in the study are included in the article/Supplementary material, further inquiries can be directed to the corresponding author.
